# Differences in *DPYD* Population Frequencies Observed in Galicians Compared to Europeans and Spanish from PhotoDPYD Study

**DOI:** 10.3390/ph18040515

**Published:** 2025-04-01

**Authors:** Almudena Gil-Rodriguez, Sheila Recarey-Rama, Ana Rodríguez-Viyuela, Raquel Cruz, Francisco Barros, Angel Carracedo, Olalla Maroñas

**Affiliations:** 1 Pharmacogenomics and Drug Discovery (GenDeM) Group, Health Research Institute of Santiago de Compostela (IDIS), 15706 Santiago de Compostela, Spain; almudena.gil@usc.es (A.G.-R.); sheila.recarey.rama@usc.es (S.R.-R.); anarovi15@gmail.com (A.R.-V.); 2 Genomic Medicine Group, CIMUS, University of Santiago de Compostela, 15782 Santiago de Compostela, Spain; raquel.cruz@usc.es (R.C.); angel.carracedo@usc.es (A.C.); 3 Centre for Biomedical Network Research on Rare Diseases (CIBERER), Instituto de Salud Carlos III, 28029 Madrid, Spain; francisco.barros@usc.es; 4 Galician Public Foundation of Genomic Medicine (FPGMX), Galician Healthcare Service (SERGAS), 15706 Santiago de Compostela, Spain; 5 Genetics Group, Health Research Institute of Santiago de Compostela (IDIS), 15706 Santiago de Compostela, Spain

**Keywords:** 5-fluorouracil, *DPYD*, allele frequency, populational databases, genetic variation

## Abstract

**Background/Objectives**: Fluoropyrimidine derivatives, 5-fluorouracil (5-FU) and its prodrugs (capecitabine and tegafur), are widely used in patients suffering from colorectal cancer. The enzyme responsible for their metabolization, dihydropyrimidine dehydrogenase (DPD), is encoded by the *DPYD* gene, which is highly polymorphic and may contain polymorphisms which could severely compromise its function. This article aims to describe the prevalence of the four main *DPYD* polymorphisms in the Galician population (Spain) and to compare these frequencies with data obtained from European cohorts in genetic databases and a Spanish study. **Methods**: Galician data frequencies for the four main *DPYD* polymorphisms recommended by the European Medicine Agency (EMA) and the Spanish Agency for Medicines and Health Products (AEMPS) (rs3918290 (c.1905+1G>A), rs55886062 (c.1679T>G), rs56038477 (c.1236G>A) and rs67376798 (c.2846A>T)) were collected, as well as data from the genomic databases 1000 Genomes and gnomAD. Additionally, the results from a Spanish *DPYD* study were included. **Results**: Significant differences in *DPYD* variant allele frequencies were observed in the Galician population compared to the frequencies reported in the European population, as well as in the Spanish PhotoDPYD study. Specifically, the rs56038477-T variant (most prevalent) along with the rs3918290-T variant, exhibited significantly lower frequencies than anticipated in the Galician cohort, with a high degree of statistical significance. **Conclusions**: Observed allele frequencies for the four *DPYD* variants suggest that Europeans and Spanish frequencies may not be fully applicable to the Galician population. These results emphasize the emerging need for incorporating the genetic information of populations that might be underrepresented into populational databases available worldwide.

## 1. Introduction

Fluoropyrimidines derivatives—specifically, 5-fluorouracil (5-FU) and its prodrugs, capecitabine and tegafur—are a group of cytostatic drugs used in oncology, especially in colorectal cancer, gastric cancer, breast cancer, and head and neck cancer [1]. These drugs are available in different formulations, 5-FU being administered as an intravenous solution and capecitabine and tegafur as oral tablets due to their condition as prodrugs. Capecitabine and tegafur, like 5-FU, are absorbed and enter the liver without any previous modification. However, they must be converted into 5-FU in order to follow the lead to be active compounds in the organism. Whereas capecitabine is transformed into 5′-deoxy-5-fluorouridine (5′dFUR) by the carboxylesterase (CES1 and CES2) and cytidine deaminase (CDA) enzymes and then converted into 5-FU by the thymidylate phosphorylase enzyme (TYMP), tegafur is converted into 5′-hydroxitegafur by the CYP2A6 enzyme and then spontaneously broken down to form 5-FU [2]. Around 80% of the 5-FU dosage is metabolized through an enzyme called dihydropyrimidine dehydrogenase (DPD), representing the rate-limiting enzyme in fluoropyrimidine catabolism and the main responsibility of the fluoropyrimidine biotransformation [3]. This step results in the origination of the dihydrofluorouracil (DHFU) metabolite, which is converted to fluoro-beta-ureidopropionate (FUPA) by the dihydropyrimidinase enzyme (DPYS) and, subsequently, to fluoro-beta-alanine (FBAL) by beta-ureidopropionase (UPB1) [2].

Although the efficacy of the fluoropyrimidines has been demonstrated in numerous clinical studies, these drugs are not without adverse reactions [4,5,6]. In fact, it is known that approximately 10% to 40% of patients develop toxicity during fluoropyrimidine treatment, leading to symptoms such as diarrhea, nausea, and leukopenia [7]. As previously mentioned, the metabolism of 5-fluorouracil (5-FU) is primarily mediated by the enzyme dihydropyrimidine dehydrogenase (DPD), which is encoded by the *DPYD* gene located on chromosome 1 at position 1p22 [8]. This gene is known to be highly polymorphic with numerous variants identified, including single nucleotide polymorphisms (SNPs) and deletions, which are cataloged in repositories such as the Pharmacogenomics Knowledge Base (PharmGKB) and Pharmacogene Variation Consortium (PharmVar). It is important to note that, unlike other pharmacogenes such as the CYP450 genes, which typically use star alleles (*) in their nomenclature, *DPYD* variants are generally named based on the position of the genetic variation [9,10].

While the *DPYD* gene exhibits significant genetic variation, it is noteworthy that only four of these variants currently have established recommendations from regulatory agencies. These variants include two that encode for a non-functioning enzyme, rs3918290 (c.1905+1G>A) and rs55886062 (c.1679T>G), as well as two others that result in reduced enzyme activity, rs56038477 (c.1236G>A) and rs67376798 (c.2846A>T). The presence of these genetic alterations, which impact the DPD enzymatic function, suggests that administering a fluoropyrimidine regimen without considering the patient’s genotype may significantly increase the risk of toxicity. This could potentially result in severe outcomes, including hospitalization in an intensive care unit (ICU) or, in extreme cases, even death [1,4,8,11,12,13,14].

In light of this situation, drug regulatory agencies, in collaboration with pharmacogenetic consortia, provide guidance on the analysis and dose adjustments for patients who carry at least one of these variants. The European Medicines Agency (EMA) recommends testing *DPYD* in patients treated with fluorouracil derivatives and suggests initiating dose reductions for carriers of specific *DPYD* variants [12]. In line with this, the Spanish Agency for Medicines and Health Products (AEMPS) also issued an informative note recommending *DPYD* analysis before initiating treatment with the four aforementioned variants [13,15,16]. The U.S. Food and Drug Administration (FDA) and Health Canada (Santé Canada) (HCSC) recommend *DPYD* testing for 5-FU and capecitabine [17,18]. Additionally, the Swiss Agency for Therapeutic Products (Swissmedic) established *DPYD* testing as mandatory for fluorouracil and actionable for capecitabine [19]. On the other hand, pharmacogenetic consortia, such as the Clinical Pharmacogenetics Implementation Consortium (CPIC^®^) and the Dutch Pharmacogenetics Working Group (DPWG), have also published prescribing guidelines with dose adjustments according to the patient’s *DPYD* genotype [5,20]. Finally, it is worth highlighting that professional societies, such as the Spanish Pharmacogenetics and Pharmacogenomics Society, the Spanish Society of Medical Oncology (SEFF/SEOM), the French National Network of Pharmacogenetics (RNPGx) and the Italian Association of Medical Oncology (AIOM), also provide recommendations based on scientific knowledge which align with CPIC^®^ guidelines [21,22,23].

In contrast, Japan’s Pharmaceuticals and Medical Devices Agency (PMDA) have classified *DPYD* testing as actionable for patients receiving fluoropyrimidine derivatives. These differences may be attributed to the extremely rare allele frequency of the identified variants within the Japanese population [24]. Thus, the distribution of allele frequency significantly influences regulatory decisions, as it directly impacts how genetic variants are interpreted in the context of health and treatment. In this regard, population databases serve as essential resources, acting as updated repositories which summarize variant information from global studies [25]. Two important genomic databases are the Genome Aggregation Database (gnomAD) [26] and the 1000 Genomes Project [27], which compile genetic variant information from genome and exome sequencing data obtained through large-scale projects across diverse populations, including the Human Genome Project and the HapMap Project [28,29]. These repositories typically aggregate genetic information from various ethnic groups and populations that share geographic characteristics, offering valuable insights into global genetic diversity. However, it is important to note that certain regions and populations may be underrepresented in these databases due to their unique idiosyncrasies, which can present challenges in fully capturing the breadth of human genetic diversity [30,31].

When it comes to genetic population differences, it is also important to make comparisons with similar studies within the same region to better contextualize the findings. In this context, the PhotoDPYD study, a cross-sectional and multicentric project conducted across different hospitals in Spain, aimed to assess the prevalence of clinically significant *DPYD* polymorphisms within the country. The study found that 4.9% of patients carried at least one *DPYD* variant, the most common of these being rs75017182 (c.1129-5923C>G), affecting 2.9% of the population. Additionally, it has been observed that a small percentage of patients were found to be compound heterozygous for *DPYD* variants. These findings highlight the genetic diversity within the population and underscore the relevance of testing for these variants in clinical settings [32].

In Galicia, a Spanish region located in the northwest part of the country, the reference genomic medicine center that provides clinical genetics services to all hospitals from the Galician Service of Health (SERGAS) is the Galician Public Foundation of Genomics Medicine (FPGMX) [33]. The FPGMX daily receives requests, including both molecular and cytogenetic analyses, from different hospitals in the Galician community through a daily internal transportation system, covering approximately 2.7 million residents [34,35].

This article aims to highlight the prevalence of the four *DPYD* polymorphisms recommended by the EMA and AEMPS in a Galician population cohort and to compare these frequencies with data frequency obtained not only from European databases, but also another cohort of the Spanish population.

## 2. Results

### 2.1. Galician Population Samples (G-Cohort)

The G-cohort is composed of 3694 patients pre-emptively genotyped, with the vast majority being wild-type for the four analyzed *DPYD* variants—rs3918290 (c.1905+1G>A), rs55886062 (c.1679T>G), rs56038477 (c.1236G>A) and rs6737698 (c.2846A>T)—and catalogued as normal metabolizers (according to the CPIC^®^ guidelines). It is worth noting that heterozygous patients carrying one reduced/non-function variant in combination with a normal function variant, as well as compound heterozygous patients were also identified, being catalogued as intermediate metabolizers. Specifically, a total of two compound heterozygous patients carrying reduced function variants, rs56038477 (c.1236G>A) and rs6737698 (c.2846A>T), were found. Although no analysis has been performed in order to determine whether these variants were in cis or trans, these patients may be at a higher risk for developing toxicity [6]. No homozygous patients for any risk allele of the four *DPYD* variants were found (Table 1).

In terms of allele frequency, the most common variants observed in G-cohort were rs56038477 (c.1236G>A) and rs6737698 (c.2846A>T), with their respective risk alleles present at 0.66% (T risk allele) and 0.64% (A risk allele). Conversely, the less common variants were rs3918290 (c.1905+1G>A) and rs55886062 (c.1679T>G), with 0.13% of patients carrying the T risk allele and 0.12% carrying the C risk allele, respectively (Table 2).

### 2.2. European and Spanish Population Cohorts

Data for the four *DPYD* polymorphisms (rs3918290 (c.1905+1G>A), rs55886062 (c.1679T>G), rs56038477 (c.1236G>A) and rs6737698 (c.2846A>T)) were collected for the three comparative cohorts. In total, allele counting was situated in 1006 alleles in 1000G-cohort, 16,180 alleles in PhotoDPYD-cohort (PhDPYD-cohort) and around 1,179,786 alleles in gnomAD-cohort (Table 3). It is important to highlight that, for the 1000G-cohort and the gnomAD-cohort, data for the rs56038477 (c.1236G>A) variant could be collected, whereas in the PhDPYD-cohort analyses the variant rs75017182 (c.1129-5923C>G). Historically, both variants were described to be in perfect linkage disequilibrium and were used indistinctly to define the HapB3 haplotype. It is worth highlighting that frequency data collected from 1000Genomes is the same for both polymorphisms, and in gnomAD frequencies are similar. In order to compare the G-cohort and the PhDPYD-cohort perfect disequilibrium was assumed.

By visual comparison of the data in both Table 2 and Table 3, it can be observed that all four variants follow similar trends in all populations, with rs56038477 (c.1236G>A) being the most prevalent and rs55886062 (c.1679T>G) being the least prevalent variants.

### 2.3. Statistical Comparisons

The χ^2^ test and Fisher’s exact test were used to compare the frequencies of the risk alleles between the four different cohorts. Statistical results, detailed in Table 4, revealed that two *DPYD* variants, rs56038477-T and rs3918290-T, exhibited significant differences between the G-cohort and the rest of the cohorts analyzed in this study. The reduced function variant rs56038477-T shows significance with p-values of 5.30 × 10^−8^ (OR = 0.28; 95% CI = 0.17–0.46), 3.60 × 10^−19^ (OR = 0.3; 95% CI = 0.23–0.40), and 2.30 × 10^−5^ (OR = 0.52; 95% CI = 0.38–0.71) when comparing the G-cohort to the 1000G-cohort, the gnomAD-cohort and the PhDPYD-cohort, respectively. In the case of the non-function variant rs3918290-T, *p*-values observed were as follows: 2.60 × 10^−2^ (OR = 0.27; 95% CI = 0.09–0.8), 1.60 × 10^−5^ (OR = 0.28; 95% CI = 0.15–0.52), and 5.20 × 10^−3^ (OR = 0.4; 95% CI = 0.2–0.78), in the same order. It should be noted that although statistical comparisons with the 1000G-cohort and the PhDPYD-cohort achieve nominal significance, they do not reach multiple correction (*p* < 0.004, provided by Bonferroni correction). The odds ratio (OR) for both variants indicate that the G-cohort variants are over 70% less frequent compared to the 1000G-cohort and the gnomAD-cohort. However, when compared to the PhDPYD-cohort, these values decrease to 60% for the rs3918290-T variant and 48% for the rs56038477-T variant. In contrast, comparisons involving the rest of the variants, rs67376798-A and rs55886062-C, did not reveal significant differences between cohorts.

## 3. Discussion

*DPYD* genetic testing is mandatory before initiating treatment with fluoropyrimidine derivatives, given its critical role in determining how patients metabolize these drugs and the potential for severe adverse effects if the testing is not conducted. Although *DPYD* is a highly polymorphic gene, only four variants currently encompass recommendations from regulatory agencies for clinical use. The aim of this article was to assess the distribution of allele frequencies for these four *DPYD* variants in the Galician population and to compare these frequencies with those observed in three different population cohorts: the European population from the 1000 Genomes Project (1000G-cohort), the non-Finnish European population from gnomAD (gnomAD-cohort), and the Spanish cohort from the PhotoDPYD study (PhDPYD-cohort), with all cohorts encompassing different population sizes.

Among the four variants analyzed, rs56038477 (c.1236G>A) was found to be the most prevalent in Galicia with an allele frequency of 0.68%. This finding was consistent with allele frequency distributions observed in the comparative cohorts and has been also reported in another European study [36]. However, the statistical comparison performed shows that although this variant was the most prevalent in the G-cohort, it is observed at a lower frequency than expected when compared with the other cohorts. Considering rs3918290 (c.1905+1G>A), identified as the third most frequent variant in the G-cohort (0.50%), the comparison revealed that this frequency is lower than expected when compared to other cohorts.

Differences between Galician and European cohorts might be explained, in part, due to the different composition in both datasets. Five different populations encompass the 1000G-cohort: CEU (Utah residents of Northern and Western European ancestry), FIN (Finnish in Finland), GBR (British in England and Scotland), IBS (Iberian populations in Spain), and TSI (Toscani in Italy), while gnomAD-cohort includes data from “British”, “White”, “Other White”, and “Irish” [37]. However, while the lack of harmonization between both cohorts could amplify statistical differences with the G-cohort, significant differences also exist with the PhDPYD-cohort, which includes, specifically, a Spanish population. Despite the presence of Galician patients in the PhotoDPYD study (data not specified), the results provided by our study presume that this population cohort might be underrepresented. It is worth highlighting that for the comparison between the G-cohort and the PhDPYD-cohort, perfect linkage disequilibrium between rs56038477 (c.1236G>A) and rs75017182 (c.1129-5923C>G) was assumed. Recent findings no longer support this assumption, and this might represent a limitation in our study [38]. From a biogeographical point of view, Galicia is situated at the westernmost point of the European continent and Spain. Some studies indicate that the Iberian Peninsula exhibits patterns of genetic differentiation, not only in comparison to other European regions but also within its own areas, showing Northern–Southern patterns, reflecting strong population migrations in medieval times [31,39,40] with Galicia and Portugal showing a more similar genetic background. It would be interesting to analyze the frequencies in Portugal and in the different regions in Spain in order to explore if the same general population genetic patterns replicate. However, the frequency of rare variants exhibits a larger variation among populations than common variation [41,42,43,44] and, in addition to this, Galicia shows an extremely high population substructure [40] that can explain our results.

Finally, the findings of this study emphasize the importance of analyzing and incorporating allele frequencies of populations that might be underrepresented in databases across different geographic regions.

## 4. Materials and Methods

### 4.1. Galician Population Samples (G-Cohort)

A total of 3694 Galician patients (2162 males and 1532 females) have been analyzed at the Pharmacogenetics Unit of the Galician Public Foundation of Genomics Medicine (FPGMX) (Santiago de Compostela, Spain) for *DPYD* since May 2020 until September 2024. These patients were directed to the Unit as a result of healthcare activity and were genotyped for the four main *DPYD* variants recommended by the AEMPs in its informative note published in May 2020 [13]: rs3918290 (c.1905+1G>A), rs55886062 (c.1679T>G), rs56038477 (c.1236G>A) and rs67376798 (c.2846A>T). Upon arrival at FPGMX, blood samples were automatically assigned with an internal code to prevent external identification. Subsequently, DNA isolation, quantification, and normalization were carried out according to the established commercial protocol. The genotyping of the samples was performed using real-time PCR technology with QuantStudio™ 5 or with an OpenArray panel on the QuantStudio™ 12K Flex (Applied Biosystems, Waltham, MA, USA). Positive and negative controls for each variant were included during the analysis. The genotyping results were analyzed using the QuantStudio™ 12K Flex Real-Time PCR Software v1.4 and the QuantStudio™ Design and Analysis Software v1.5.2, along with the Thermo Fisher Cloud Solution [45].

### 4.2. Data Selection of European and Spanish Populations

In order to compare the allele frequency distribution of the four major *DPYD* polymorphisms between the Galician population (G-cohort) and the European population, gnomAD [21] and 1000 Genomes [22] databases were explored. Specifically, in the 1000 Genomes Project, the European population cohort was selected using GRCh38.p14 as a reference genome, forming the 1000G-cohort. In the case of gnomAD, the non-Finnish population cohort was selected (as the European population is divided into Finnish and non-Finnish), with GRCh38 version v4.1 serving as the reference genome for this cohort (gnomAD-cohort). In addition, a Spanish subset of results from the PhotoDPYD project (PhDPYD-cohort) was included in the analysis [27].

### 4.3. Statistical Analysis

In order to perform statistical associations between the genotypic frequencies of the G-cohort and the population datasets selected, two statistical methods were employed: the χ^2^ test and Fisher’s exact test. If the smallest cell count was equal to or less than five, Fisher’s exact test was chosen, and the exact two-tailed probability was reported as a measure of significance. To complement the comparison, odds ratios (OR) were calculated to assess the strength of the association between the variables. To account for the number of tests performed (4 SNPs and three comparisons with European populations), a Bonferroni correction was applied, adjusting for 12 tests. Therefore, the significance level was set at 0.004 (0.05/12). All statistical analyses were performed using R software (version 4.1.1).

## 5. Conclusions

The current study reveals that frequency values for *DPYD* variants rs55886062 (c.1679T>G) and rs67376798 (c.2846A>T) align between the three populations compared (Galicians, Europeans and Spanish). This suggests that there are no significant differences in the frequency of these variants between these populations, indicating a common distribution regarding the prevalence of these genetic variants. However, statistically significance differences were observed for the variants rs56038477 (c.1236G>A) and rs3918290 (c.1905+1G>A), which suggest that while the allele frequencies of *DPYD* variants observed in Europeans and in the Spanish population may be similar, they may not be fully representative of the Galician population. Therefore, the results presented in this article emphasize the emerging need to incorporate genetic information of populations that might be underrepresented into global populational databases available worldwide. This is essential for a more accurate and comprehensive understanding of genetic variability, taking into account the specificities of particular population groups.

## Figures and Tables

**Table 1:** G-cohort *DPYD* genotype distribution per variant.

	rs3918290	rs55886062	rs56038477	rs67376798
Wild-type	3684 (99.73%)	3685 (99.76%)	3644 (98.65%)	3646 (98.70%)
Heterozygous	10 (0.27%)	9 (0.24%)	50 (1.35%)	48 (1.30%)

**Table 2:** G-cohort *DPYD* allele frequency distribution per variant.

Variant	G-Cohort
rs3918290-C	7378 (99.86%)
rs3918290-T*	10 (0.14%)
rs55886062-A	7379 (99.86%)
rs55886062-C*	9 (0.12%)
rs56038477-A	7338 (99.32%)
rs56038477-T*	50 (0.68%)
rs67376798-T	7340 (99.35%)
rs67376798-A*	48 (0.65%)

*—risk allele of the variant; G-cohort—Galician FPGMX cohort. The percentage included represents the number of alleles over the total.

**Table 3:** Number of variant alleles for each population cohort.

Variant	1000G-Cohort	gnomAD-Cohort	PhDPYD-Cohort
rs3918290-C	1001 (99.5%)	1,174,085 (99.52%)	16,053 (99.66%)
rs3918290-T*	5 (0.50%)	5701 (0.48%)	55 (0.34%)
rs55886062-A	1005 (99.9%)	1,178,286 (99.92%)	16,093 (99.91%)
rs55886062-C*	1 (0.10%)	996 (0.08%)	15 (0.09%)
rs56038477-A	982 (97.61%)	1,153,662 (97.79%)	-
rs56038477-T*	24 (2.39%)	26,048 (2.21%)	-
rs75017182-G	-	-	15,899 (98.70%)
rs75017182-C*	-	-	209 (1.30%)
rs67376798-T	999 (99.3%)	1,171,791 (99.36%)	16,003 (99.35%)
rs67376798-A*	7 (0.7%)	7853 (0.64%)	105 (0.65%)

*—risk allele of the variant; 1000G-cohort—1000 Genomes European cohort; gnomAD-cohort—gnomAD European cohort (non-Finnish population); PhDPYD-cohort—PhotoDPYD study cohort. The percentage included represents the number of alleles over the total.

**Table 4:** Results from the statistical comparative analysis.

**Variant**	**G-Cohort**	**1000G-Cohort**	**OR (95% CI)**	***p* Value**
*rs3918290-T*	*10 (0.14%)*	*5 (0.50%)*	*0.27 (0.09–0.8)*	*2.60 × 10^−2^*
rs55886062-C	9 (0.12%)	1 (0.10%)	1.23 (0.16–9.69)	1.0
rs56038477-T	50 (0.68%)	24 (2.39%)	0.28 (0.17–0.46)	5.30 × 10^−8^
rs67376798-A	48 (0.65%)	7 (0.7%)	0.93 (0.42–2.07)	8.60 × 10^−1^
**Variant**	**G-cohort**	**gnomAD-cohort**	**OR (95% CI)**	***p* Value**
rs3918290-T	10 (0.14%)	5701(0.49%)	0.28 (0.15–0.52)	1.60 × 10^−5^
rs55886062-C	9 (0.12%)	996(0.08%)	1.44 (0.75–2.78)	2.70 × 10^−1^
rs56038477-T	50 (0.68%)	26,048 (2.16%)	0.3 (0.23–0.40)	3.60 × 10^−19^
rs67376798-A	48 (0.65%)	7853(0.64%)	1.01 (0.76–1.34)	9.40 × 10^−1^
**Variant**	**G-cohort**	**PhDPYD-cohort**	**OR (95% CI)**	***p* Value**
*rs3918290-T*	*10 (0.14%)*	*55 (0.34%)*	*0.4 (0.20–0.78)*	*5.20 × 10^−3^*
rs55886062-C	9 (0.12%)	15 (0.09%)	1.31 (0.57–2.999	5.20 × 10^−1^
rs56038477-T	50 (0.68%)	209 (1.30%)	0.52 (0.38–0.71	2.30 × 10^−5^
rs67376798-A	48 (0.65%)	105 (0.65%)	1 (0.71–1.40)	9.80 × 10^−1^

G-cohort—Galician FPGMX cohort; 1000G-cohort—1000 Genomes European cohort; gnomAD-cohort—gnomAD European cohort (non-Finnish population); PhDPYD-cohort—PhotoDPYD study. The percentage included represents the number of alleles over the total. OR—odds ratio; CI—confidence interval. Italic *p* values indicate nominal significance without achieving Bonferroni threshold (*p* < 0.004).

## Data Availability

The data analyzed in this study is subject to the following licenses/restrictions: Data derived from clinical practice. Requests to access these datasets should be directed to Olalla Maroñas, olalla.maronas@usc.es.

## Abbreviations

The following abbreviations are used in this manuscript:

5-FU	5-Fluorouracil
DPD	Dihydropyrimidine dehydrogenase
5′dFUR	5′-Deoxy-5-fluorouridine
CES1 and CES2	Carboxylesterase
CDA	Cytidine deaminase
TYMP	Thymidylate phosphorylase enzyme
DHFU	Dihydrofluorouracil
FUPA	Fluoro-beta-ureidopropionate
DPYS	Dihydropyrimidinase enzyme
FBAL	Fluoro-beta-alanine
UPB1	Beta-ureidopropionase
EMA	European Medicines Agency
PharmGKB	Pharmacogenetics and Pharmacogenomics Knowledge Base
PharmVar	Pharmacogene Variation Consortium
AEMPS	Spanish Agency for Medicines and Health Products
FDA	Food and Drug Administration
HCSC	Health Canada (Santé Canada)
Swissmedic	Swiss Agency for Therapeutic Products
CPIC^®^	Clinical Pharmacogenetics Implementation Consortium
DPWG	Dutch Pharmacogenetics Working Group
SEFF/SEOM	Pharmacogenomics Society and the Spanish Society of Medical Oncology
RNPGx	French National Network of Pharmacogenetics
AIOM	Italian Association of Medical Oncology
PMDA	Japan’s Pharmaceuticals and Medical Devices Agency
ICU	Intensive Care Unit
gnomAD	Genome Aggregation Database
FPGMX	Pharmacogenetic Unit of the Public Foundation of Genomic Medicine
SERGAS	Galician Service of Health
G-cohort	Galician FPGMX cohort
1000G-cohort	1000 Genomes European cohort
gnomAD-cohort	gnomAD European cohort (non-Finnish population)
PhDPYD-cohort	PhotoDPYD study cohort
CEU	Utah residents of Northern and Western European ancestry
FIN	Finnish in Finland
GBR	British in England and Scotland
IBS	Iberian populations in Spain
TSI	Toscani in Italy
OR	Odds ratio
